# The Generation of iPSCs Expressing Interferon-Beta Under Doxycycline-Inducible Control

**DOI:** 10.3390/ijms26178376

**Published:** 2025-08-28

**Authors:** Olga Sheveleva, Nina Butorina, Elena Protasova, Sergey Medvedev, Elena Grigor’eva, Victoria Melnikova, Valeriia Kuziaeva, Marina Minzhenkova, Yana Tatarenko, Irina Lyadova

**Affiliations:** 1 Laboratory of Cellular and Molecular Basis of Histogenesis, Koltzov Institute of Developmental Biology of the Russian Academy of Sciences, Moscow 119334, Russia; nnbut@mail.ru (N.B.);; 2 Laboratory of Developmental Epigenetics, Institute of Cytology and Genetics, Siberian Branch of Russian Academy of Sciences, Novosibirsk 630090, Russia; medvedev@bionet.nsc.ru (S.M.); evlena@bionet.nsc.ru (E.G.); 3 Laboratory of Comparative Developmental Physiology, Koltzov Institute of Developmental Biology of the Russian Academy of Sciences, Moscow 119334, Russia; v_melnikova@mail.ru; 4 Laboratory of Cell Biology, Koltzov Institute of Developmental Biology of the Russian Academy of Sciences, Moscow 119334, Russiaanka92-09@bk.ru (Y.T.)

**Keywords:** induced pluripotent stem cells, interferon-beta (IFN-β), CRISPR/Cas9 technology, doxycycline-inducible gene expression, trilineage differentiation

## Abstract

Type 1 interferons (IFN-Is) exhibit significant antiviral, antitumor, and immunoregulatory properties, demonstrating substantial therapeutic potential. However, IFN-Is are pleiotropic cytokines, and the available data on their effect under specific pathological conditions are inconclusive. Furthermore, the systemic administration of IFN-Is can result in side effects. Generating cells that can migrate to the pathological focus and provide regulated local production of IFN-Is could overcome this limitation and provide a model for an in-depth analysis of the biological and therapeutic effects of IFN-Is. Induced pluripotent stem cells (iPSCs) are a valuable source of various differentiated cell types, including human immune cells. In this study, we describe the generation of genetically modified human iPSCs with doxycycline-controlled overexpression of interferon β (IFNB1). Three *IFNB1*-overexpressing iPSC lines (IFNB-iPSCs) and one control line expressing the transactivator M2rtTA (TA-iPSCs) were generated using the CRISPR/Cas9 technology. The pluripotency of the generated cell lines has been confirmed by the following: (i) cell morphology; (ii) the expression of the pluripotency markers OCT4, SOX2, TRA 1-60, and NANOG; and (iii) the ability to spontaneously differentiate into the derivatives of the three germ layers. Upon the addition of doxycycline, all IFNB-iPSCs upregulated *IFNB1* expression at RNA (depending on the iPSC line, 126-816-fold) and protein levels. The IFNB-iPSCs and TA-iPSCs generated here represent a valuable cellular model for studying the effects of IFN-β on the activity and differentiation trajectories of different cell types, as well as for generating different types of cells with controllable IFN-β expression.

## 1. Introduction

Induced pluripotent stem cells (iPSCs) can be differentiated into various types of somatic cells of the living organism; they demonstrate high expansion under in vitro conditions, and they are well amenable to genetic modifications [1,2,3]. In this regard, iPSCs have been successfully used as a model to study early developmental processes, as well as to model pathologies, and to decipher the biological role of individual genes in organism development and cell functioning [3,4,5]. In the latter case, the main approaches usually involve either deleting the target gene or introducing the mutation associated with a particular pathology [6,7,8]. There are only few studies that have analyzed how overexpression of a certain gene affects the biology of iPSCs and their progeny, which limits our understanding of the biological role of these genes [9,10,11,12].

Type 1 interferons (IFN-Is), including interferon β, are cytokines that play a key role in antiviral immunity and participate in antibacterial and antifungal immunity [13,14,15,16]. In addition, IFN-Is show antitumor activity [17]. In this regard, the possibility of using IFN-Is and/or their inducers as part of a comprehensive therapy of infectious and tumor diseases is being actively investigated [18,19,20]. At the same time, systemic administration of interferons may lead to side effects, including anorexia, depression, hepatotoxicity, and others [21,22,23]. Therefore, it is of interest to develop approaches that can provide localized and controlled secretion of IFN-Is. For this purpose, a model based on obtaining genetically modified iPSCs with inducible (doxycycline-controlled) overexpression of IFN-Is and their subsequent differentiation into immune cells is very appropriate. The feasibility and safety of using cells generated from iPSCs are being actively investigated in a series of clinical trials and have yielded encouraging results [24].

Obtaining iPSCs overexpressing IFN-Is is also of interest as a platform for studying various biological effects of interferons, including the influence of IFN-Is on the processes of early development and differentiation of human cells, which are difficult to model using other approaches [25,26]. Recently, we have obtained human iPSC lines with the constitutive overexpression of IFN-β, the use of which revealed IFN-dependent impairments of iPSC neuroectodermal differentiation [26]. However, the constant expression of IFN-β during the differentiation did not allow us to identify critical stages at which IFN-β affected that affect cell differentiation.

In view of the above, in this study, we set out to obtain iPSC lines with doxycycline-controlled IFN-β overexpression. Here, we describe the generation and detailed characterization of three human iPSC lines with doxycycline-inducible *IFNB1* expression. These iPSC lines can be used to generate different cell types with controlled IFN-β expression.

## 2. Results

**Figure 1:**
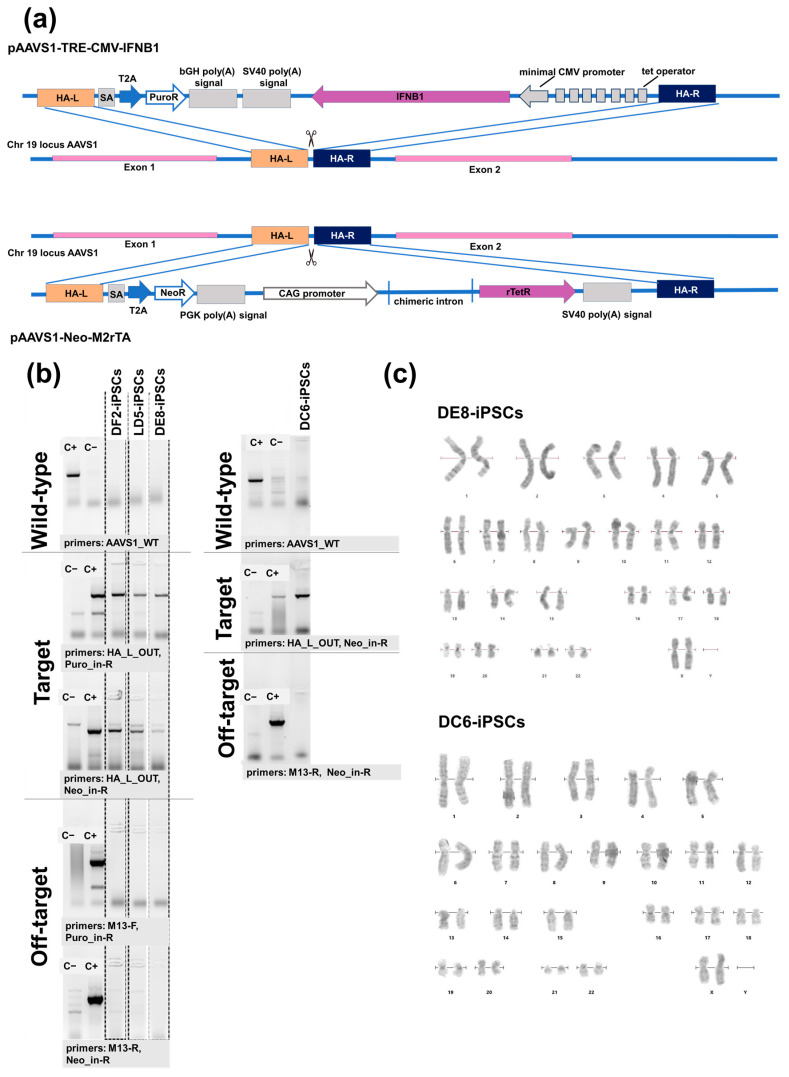
Generation of doxycycline-inducible *IFNB1*-overexpressing human iPSC lines (IFNB-iPSCs) and a control line expressing the transactivator *M2rtTA* (TA-iPSCs): (**a**) Schematic representation of transgenesis at the AAVS1 locus. The plasmids used to modify parental K7-iPSCs were pAAVS1-TRE-CMV-IFNB1, containing the target *IFNB1* gene under a doxycycline-driven promoter, and AAVS1-Neo-M2rtTA, containing the *M2rtTA* transactivator (see the Supplement, Figure S1 for plasmid maps); (**b**) Screening for on-target and off-target insertions in genetically modified IFNB-iPSCs (DF2-, LD5-, and DE8-iPSCs) and TA-iPSCs (DC6-iPSCs). Wild-type: The presence of unmodified AAVS1 loci was assessed using parental K7-iPSCs as a positive control (C+) and a previously generated iPSC line carrying a verified correct insert of the roGFP2-Orp1 transgene [29] as a negative control (C−). Target: The presence of an insert at the AAVS1 locus was evaluated using the iPSC line with a verified correct insert of the roGFP2-Orp1 transgene at the AAVS1 locus [29] as a positive control (C+) and parental K7-iPSCs as a negative control (C−). Off-target: Off-target integration was tested using the plasmids pAAVS1-TRE-CMV-IFNB1 and pAAVS1-Neo-M2rTA as positive controls (C+) and parental K7-iPSCs as a negative control (C−). Primers used for each analysis are indicated in the Figure and listed in Table 1. (**c**) Karyograms of DE8- and DC6-iPSCs (karyograms of LD5-IPSCs and DF2-iPSCs are presented in the Supplementary Figure S2).

Human iPSCs of the K7-4Lf line (registered as ICGi022-A [27]; hereafter referred to as K7-iPSCs) were previously derived from peripheral blood mononuclear cells of a healthy donor. The cells were modified by electroporation using three plasmids: (1) pAAVS1-TRE-CMV-IFNB1 carrying the target gene (Supplementary Figure S1a), (2) pAAVS1-Neo-M2rtTA carrying the gene encoding the *M2rtTA* transactivator (Supplementary Figure S1b), and (3) pX458-AAVS1 plasmid carrying elements of the CRISPR/Cas9 system, as described in “Materials and Methods” (Figure 1a). Following puromycin/neomycin selection, 16 lines were established. The lines were expanded and screened for the presence of on-target insertions and the absence of off-target integrations of plasmid DNA carrying the target gene (Figure 1b). Off-target effects of the AAVS1-targeting sgRNA encoded by the pX458-AAVS1 plasmid have been previously evaluated using the same parental K7-iPSC line as in the present study and were not detected [28]. As a result, three lines were chosen for further analysis based on the results of PCR analysis for target insertions, cell morphology and growth rate: DE8-iPSCs, LD5-iPSCs, and DF2-iPSCs (Figure 1b). In parallel with these lines, a control line expressing the *M2rtTA* transactivator without *IFNB1* was generated: DC6-iPSCs.

**Figure 2:**
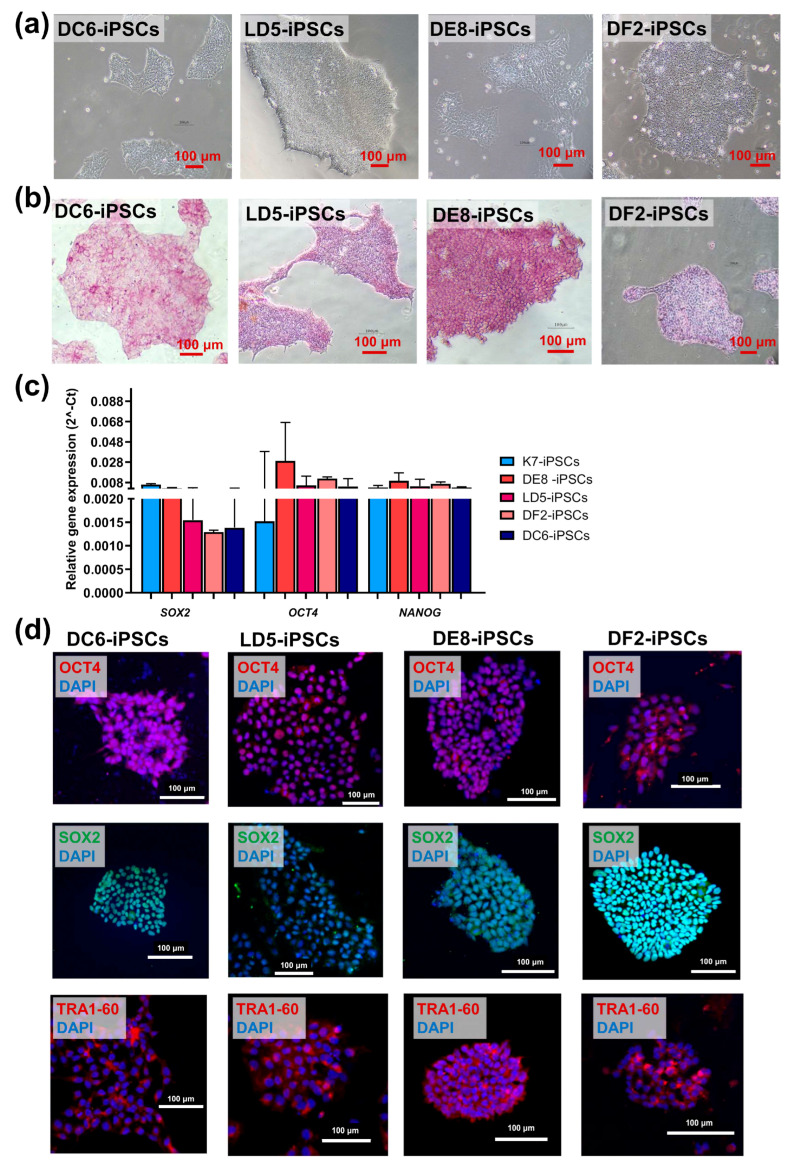
IFNB-iPSCs (line LD5, DE8, DF2) and TA-iPSCs (line DC6) display normal morphology and expression of pluripotency markers: (**a**) the morphology of IFNB-iPSCs and TA-iPSCs; light microscopy, phase contrast; (**b**) IFNB-iPSCs and TA-iPSCs are positively stained for alkaline phosphatase. (**c**,**d**) IFNB-iPSCs and TA-iPSCs express pluripotency markers SOX2, OCT4, NANOG and TRA-1-60 at a level comparable with parent K7-iPSCs: (**c**) RT-qPCR; and (**d**) immunostaining and confocal microscopy (the nuclei are stained with DAPI, blue). In all images, the scale bar is 100 µm.

All four lines had a normal 46XX karyotype (Figure 1c), displayed a morphology characteristic of human iPSCs (Figure 2a), and were positive for alkaline phosphatase (Figure 2b).

The expression levels of the genes encoding the pluripotency markers *OCT4*, *SOX2*, *NANOG* were comparable with those in control parental K7-iPSCs (Figure 2c). Immunocytochemical staining of colonies of DE8-iPSCs, LD5-iPSCs, DF2-iPSCs, and DC6-iPSCs demonstrated the expression of pluripotency markers OCT4, SOX2, and TRA-1-60 at the protein level (Figure 2d).

**Figure 3:**
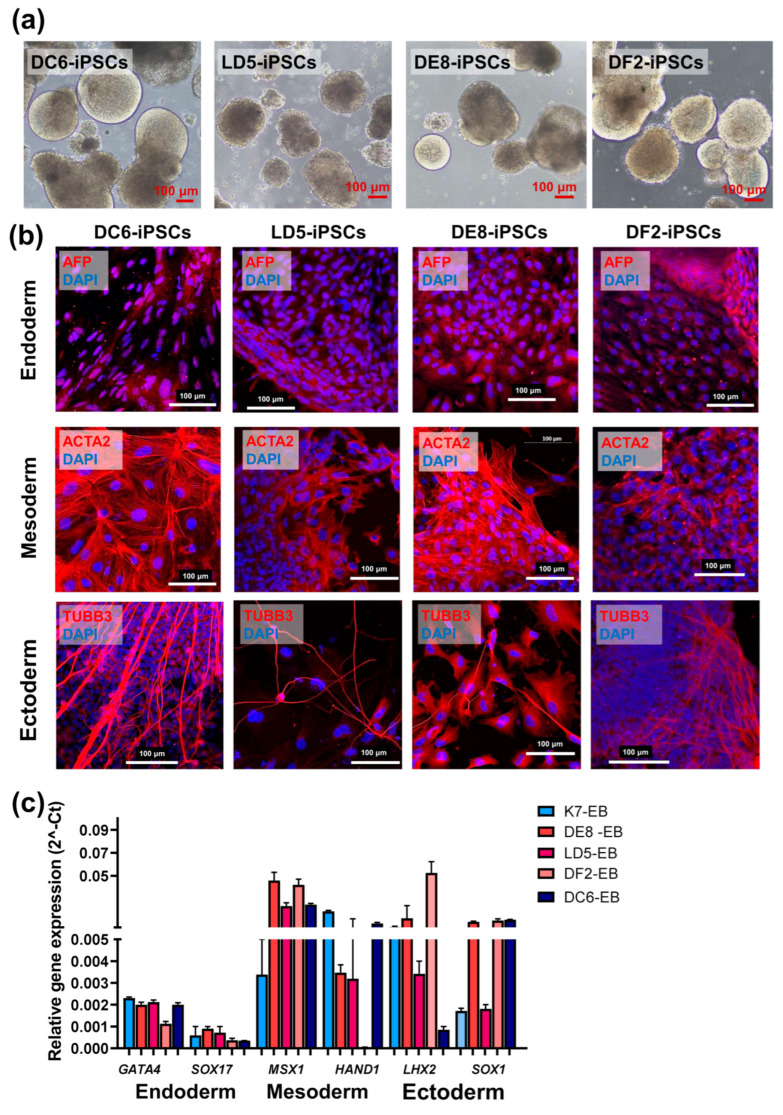
IFNB-iPSCs (line LD5, DE8, DF2) and TA-iPSCs (line DC6) differentiate into the derivatives of the three primary germ layers (in all images, the scale bar is 100 µm): (**a**) Embryoid bodies (EBs) spontaneously generated from IFNB-iPSCs and TA-iPSCs-light microscopy, phase contrast; (**b**) The expression of TUBB3 (ectoderm), AFP (endoderm), and ACTA2 (mesoderm) visualized by immunostaining and confocal microscopy (the nuclei are stained with DAPI, blue); (**c**) The expression of *GATA4*, *SOX17* (endoderm), *MSX1*, *HAND1* (mesoderm), and *LHX2*, *SOX1* (ectoderm) analyzed by RT-qPCR.

To characterize the differentiation potencies of the generated iPSC lines, embryoid bodies were obtained and were cultured for 20 days, as described in “Materials and Methods” (Figure 3a). Expression analysis for the main markers of the derivatives of the three germ layers, both at the mRNA and protein levels, showed that all the investigated lines possess the characteristic differentiation potencies of iPSCs similar to the parental line (K7-iPSCs) (Figure 3b,c).

**Figure 4:**
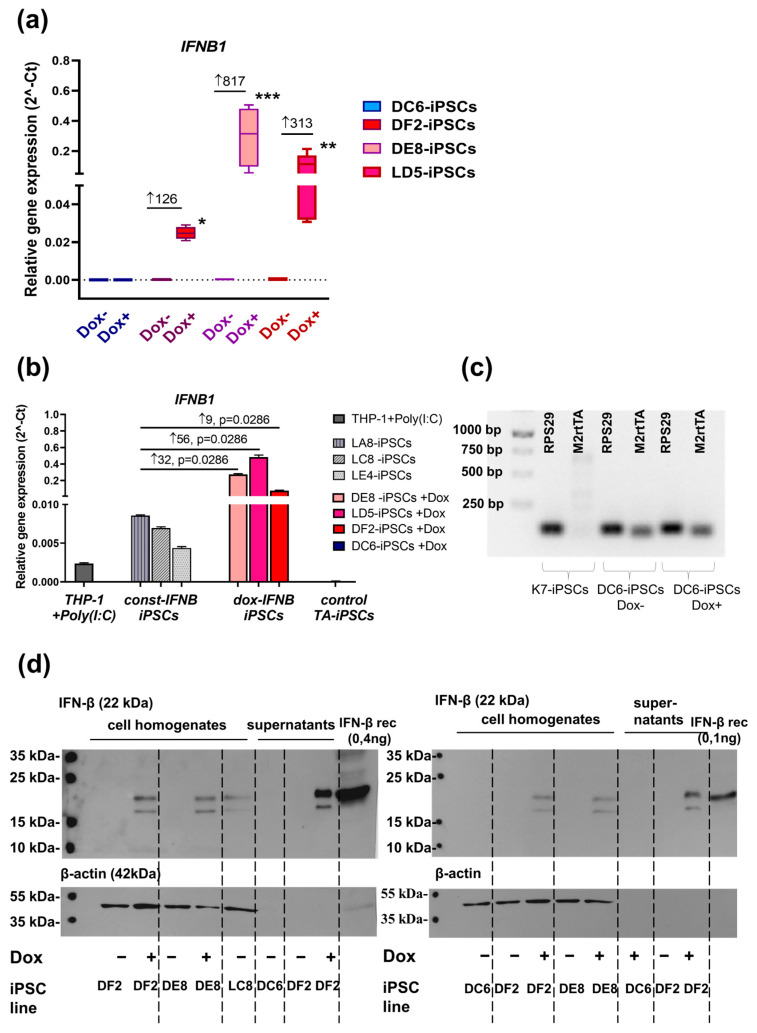
IFNB-iPSCs display doxycycline-inducible expression of *IFNB1*: (**a**) IFNB-iPSCs cultivated with doxycycline (Dox) for 24 h overexpress *IFNB1*, as compared with doxycycline-unstimulated cells and control TA-iPSCs (line DC6). *IFNB1* expression values were normalized relative to *RPS29*, Mann–Whitney test, * *p* < 0.05, ** *p* < 0.01, *** *p* < 0.001; ↑, fold increase relative to Dox-. (**b**) Doxycycline-stimulated IFNB-iPSCs express *IFNB1* at higher levels (↑) than iPSCs with the constitutive *IFNB1* expression (const-IFNB-iPSCs) and than THP-1 stimulated with Poly (I:C). *IFNB1* expression values were normalized relative to *RPS29*. LA8, LC8, LE4 const-IFNB-iPSCs line. Mann–Whitney test. ↑, fold increase relative to the indicated const-IFNB-iPSCs. (**c**) DC6-iPSCs express the transactivator *M2rtTA* unlike parental K7-iPSCs (RT-PCR, agarose gel); (**d**) IFNB-iPSCs stimulated with doxycycline for 24 h produce IFN-β. Western blotting was performed using: (i) homogenates of DF2 and DE8 IFNB-iPSCs; (ii) homogenates of DC6 TA-iPSCs; (iii) supernatants of DF2 IFNB-iPSCs (all-two experiments); (iv) homogenates of LC8 const-IFNB-iPSCs; and (v) supernatants of DC6 TA-iPSCs (one experiment). IFNB-iPSCs and TA-iPSCs were either unstimulated or stimulated with doxycycline (2 µg/mL, 24 h). Recombinant IFN-β was used as a positive control and was added in different amounts in the two experiments, as indicated. Cell lysates were normalized to β-actin.

To confirm the inducible expression of the target gene (*IFNB1*) in IFNB-iPSCs, doxycycline (2 μg/mL, Abcam, Cambridge, UK) was added to the iPSC cultures. Twenty-four hours later, the expression of *IFNB1* was determined using RT-PCR. Compared with IFNB-iPSCs cultured without doxycycline, IFNB-iPSCs stimulated with doxycycline (dox-IFNB-iPSCs) increased *IFNB1* expression 126–816-fold on average (*p*-values for DF2-iPSCs, DE8-iPSCs and LD5-iPSCs were 0.0286, 0.0006 and 0.0079, respectively, Figure 4a).

Recently, we have generated human iPSC lines with the constitutive overexpression of *IFNB1* (const-IFNB-iPSCs) [26]. To evaluate how efficiently doxycycline upregulated *IFNB1* expression in IFNB-iPSCs generated in the current study, we next compared the levels of *IFNB1* expression in: (i) three lines of IFNB-iPSCs stimulated with doxycycline; (ii) three lines of const-IFNB-iPSCs; and (iii) THP-1 cells stimulated with polyinosinic-polycytidylic acid (i.e., poly (I:C), a synthetic analogue of viral double-stranded RNA, a potent inducer of IFN-β. In all three IFNB-iPSC lines tested, the levels of *IFNB1* expression were significantly (*p* < 0.03) higher than in the groups of comparison (Figure 4b).

No increase in *IFNB1* expression in response to doxycycline was observed in the control DC6-iPSC line. RT-PCR analysis showed that the *M2rtTA* transactivator was stably expressed in DC6-iPSC line, and its expression did not depend on cell stimulation with doxycycline (comparison with the control parental K7-iPSC line, Figure 4c).

To check whether IFNB-iPSCs expressed IFN-β at protein level, we performed Western blotting of cell homogenates. Cell homogenates were obtained from: (i) DF2 and DE8 IFNB-iPSCs, either unstimulated or doxycycline-stimulated; (ii) DC6 TA-iPSCs, treated or untreated with doxycycline; and (iii) LC8 const-IFNB-iPSCs. We observed IFN-β production in dox-IFNB-iPSCs and unstimulated const-IFNB-iPSCs, but we did not detect IFN-β in unstimulated IFNB-iPSCs nor in the control DC6-iPSCs (either doxycycline-stimulated or unstimulated). Since IFN-β is a secreted protein, we also examined cell culture supernatants and detected IFN-β in the supernatants of doxycycline-stimulated IFNB-iPSCs (line DF2, Figure 4d).

Overall, the newly generated IFNB-iPSC lines retained all key features of pluripotency and expressed IFN-β at both RNA and protein levels in a doxycycline-inducible manner.

## 3. Discussion

In recent years, in addition to the immunological role of IFN-Is, researchers have become interested in the impact of IFN-Is on cell differentiation processes, IFN-I involvement in neuroinflammation and in the development of neurodegenerative diseases such as Alzheimer’s and Parkinson’s, as well as in their role in embryonic development [30,31,32,33,34]. For these studies, especially those concerning the effect of IFN-Is on early human development, an adequate cellular model is needed.

Recently, Eggenberg and co-authors analyzed how the induced expression of IRF7, the transcriptional factor involved in IFN-I response, affects the biology of human iPSCs. The authors found that IRF7 expression impairs iPSC differentiation into ectodermal and endodermal directions [25]. The results suggested a role for IFN-Is in the negative regulation of early developmental processes, which is an important observation since (i) IFN-Is are abundantly produced during infections, particularly, viral and (ii) viral infections are known to hamper neuronal development [35,36]. However, the study by Eggenberg and co-authors investigated the effects of IFN-Is indirectly through the induction of a regulatory factor. Given that IRF7 plays a role not only in the induction of IFN-Is but also in other cellular responses, such as cell growth and apoptosis [37], this limited the interpretation of the study results. More recently, we generated human iPSCs with the constitutive expression of *IFNB1* and revealed a direct inhibitory effect of IFN-β overexpression on the differentiation of iPSCs into neuroectoderm [26]. However, our study also had limitations since the constitutive overproduction of IFN-β by IFNB-iPSCs did not allow to identify critical developmental points at which increased levels of IFN-β could lead to negative consequences for neuroectoderm development. Taking this into account, in this study, we aimed to generate iPSC lines with the inducible expression of *IFNB1*. Results presented show document the possibility of producing human iPSC lines with doxycycline-controlled overexpression of *IFNB1*. The three lines of IFNB-iPSCs we obtained show a significant increase in the expression of the target *IFNB1* gene upon the addition of doxycycline and meet all the criteria for human iPSCs. IFN-β protein production was detected in both cell lysates and supernatants obtained from dox-IFNB-iPSCs. In the future, these IFNB-iPSCs can be used to study the effects of IFN-Is on cell differentiation, embryonic development, and the pathogenesis of IFN-I-associated diseases, as well as to identify targets for the treatment of such diseases.

## 4. Materials and Methods

### 4.1. Cultivation of iPSCs

Parental iPSCs of the K7-4LF line (hereinafter, K7-iPSCs) obtained from the peripheral blood monocytes of a healthy donor and kindly provided to us for research by the Laboratory of Developmental Epigenetics at the Institute of Cytology and Genetics of the Siberian Branch of the Russian Academy of Sciences [27], were cultured on matrigel (Corning, Somerville, MA, USA) in mTeSR™1 medium (StemCell Technologies, Vancouver, BC, Canada) supplemented with 1% penicillin/streptomycin (HyClone, Logan, UT, USA) or on mouse embryonic fibroblasts (MEFs) in KnockOut™ DMEM medium (Thermo Fisher Scientific, Waltham, MA, USA) supplemented with: 15% KnockOut™ Serum Replacement (Thermo Fisher Scientific, Waltham, MA, USA), 1% substituted amino acid blend (Gibco™/Thermo Fisher Scientific, Carishbad, CA, USA), 1% GlutaMAX supplement (Thermo Fisher Scientific, Waltham, MA, USA), and 10 ng/mL basic fibroblast growth factor (bFGF, SCI Store, Moscow, Russia). iPSCs were passaged when 70–80% confluency was reached, using the TrypLE reagent (Thermo Fisher Scientific, Waltham, MA, USA), and were cultured in a fresh medium for iPSCs, with the addition of 10 μM ROCK inhibitor thiazovivin (Sigma-Aldrich, St. Louis, MO, USA) one day after passaging.

### 4.2. Obtaining iPSCs with IFN-β Overexpression

10^6^ K7-iPSCs were transfected with an equimolar mixture of three donor plasmids (5 μg in total) using a 100 μL Neon™ Transfection System kit (Invitrogen™, Waltham, MA, USA) and a Neon™ electroporator (Thermo Fisher Scientific, Waltham, MA, USA), with the following program: 1650 V; 10 ms; 3 pulses. The following plasmids were used for the electroporation of K7-iPSCs: (1) pAAVS1-TRE-CMV-IFNB1, encoding the target *IFNB1* gene under a doxycycline-driven promoter, which is activated by a transactivator protein upon its binding to doxycycline, as well as the puromycin resistance gene (Figure 1a); (2) AAVS1-Neo-M2rtTA, encoding a reverse transactivator for doxycycline-controlled expression and a neomycin resistance gene (plasmid #60843, Addgene, Watertown, MA 02472, USA) (Figure 1a); and (3) pX458-AAVS1, encoding SpCas9 nuclease, EGFP, and AAVS1 sgRNA (based on pSpCas9 (BB)-2A-GFP (plasmid #48138, Addgene, Watertown, MA 02472, USA). In parallel with the IFNB1 overexpressing lines, a control iPSC line containing the transactivator gene but without the target *IFNB1* gene was obtained under the same conditions. After electroporation, iPSCs were cultured in 3.5 cm Petri dishes coated with MEFs in iPSC medium supplemented with ROCK inhibitor. The selection of mutant iPSCs resistant to the antibiotics neomycin and puromycin was started the day after transfection. The antibiotics (300 ng/mL for puromycin, AppliChem, Council Bluffs, IA, USA, and 50 μg/mL for geneticin (neomycin) G-418, Thermo Fisher Scientific, Waltham, MA, USA) were added to penicillin/streptomycin-free medium sequentially, culturing the cells for 4 days in the presence of puromycin and then for 4 days in the presence of geneticin. Sixteen cell lines that survived the selection procedures were obtained, expanded and tested for the presence of on-target and off-target effects. For that, genomic DNA was extracted from the growing clones using the QuickExtract™ kit (Lucigen, Madison, WI, USA) and amplified in PCR to detect: (i) the wild-type AAVS1 locus lacking the target insert; (ii) the modified AAVS1 locus containing the target insert; and (iii) randomly incorporated segments of the donor plasmid. The primers used for the analysis were as follows: (i) AAVS1_WT-F and AAVS1_WT-R; (ii) HA_L_OUT and Puro_in-R and HA_L_OUT and Neo_in-R; and (iii) M13-F and Puro_in-R and Neo_in-R and M13-R. Primer sequences are listed in Table 1. PCR products were analyzed by polyacrylamide gel electrophoresis.

### 4.3. Karyotype Analysis

Karyotyping was performed at the Collective Use Center of the Koltzov Institute of Developmental Biology of the Russian Academy of Sciences. For karyotyping, iPSCs were grown on 3.5 cm Petri dishes covered with matrigel. When the cells reached 70–80% confluency, they were treated with colcemid (0.1 μg/mL, Biolot, Saint-Petersburg, Russia) for 1 h at 37 °C. The cells were then washed with PBS, dissociated using 0.25% trypsin/EDTA solution (3 min, Gibco™/Thermo Fisher Scientific, Carishbad, CA, USA), and incubated in hypotonic KCl solution (0.56 M, 37 °C, 20 min). For prefixation, 2–3 drops of Carnoy’s solution (methanol:acetic acid, 3:1) were added to the KCl solution. The cells were gently resuspended, precipitated (1400 rpm, 7 min), and incubated in ice-cold Carnoy’s solution (15 min on ice), after which the cycle was repeated twice. The preparations were stained with 5% Giemsa solution for 10–20 min. G-stained chromosomes were analyzed using the International System for Human Cytogenomic Nomenclature. At least 20 metaphase spreads were analyzed.

### 4.4. Alkaline Phosphatase Staining

The detection of endogenous alkaline phosphatase in iPSCs was performed using the Leukocyte Alkaline Phosphatase kit (Sigma-Aldrich, St. Louis, MO, USA) according to the manufacturer’s protocol. The cells were washed with PBS, fixed with citrate-acetone-formaldehyde fixative, washed, stained with alkaline dye mixture (15 min in the dark), and washed with deionized water.

### 4.5. Mycoplasma Detection

To detect mycoplasma contamination, the obtained iPSC lines were cultured under normal conditions on matrigel-coated plates. After reaching 80% confluency, the cells were removed with lysing buffer and genomic DNA was isolated using the Extract DNA kit according to the manufacturer’s instructions (Evrogen, Moscow, Russia). PCR with genomic DNA was performed with primers to the ribosomal RNA gene (Table 1) under the following conditions: 95 °C—3 min; 35 cycles: 95 °C—15 s, 67 °C—15 s, 72 °C—20 s, and 72 °C—5 min. All the lines were negative for mycoplasma contamination.

**Table 1:** Primers used in PCR and RT-qPCR.

Target	Forward/Reverse Primer (5′-3′)
*OCT4*	GGGTTCTATTTGGGAAGGTATT/GGGTTTCTGCTTTGCATATCT
*NANOG*	GAAATACCTCAGCCTCCAGC/TGCCACCTCTTAGATTTCATTC
*SOX2*	CTCGCAGACCTACATGAACG/GAGCCAAGAGCCATGCC
*IFNB1*	GAACTTTGACATCCCTGAGGAG/CCAGTGCTAGATGAATCTTGTCTG
GATA4	GCGGTGCTTCCAGCAACTCCA/GACATCGCACTGACTGAGAACG
SOX17	ACGCTTTCATGGTGTGGGCTAAG/GTCAGCGCCTTCCACGACTT
MSX1	GCGCCAAGGCAAAGAGAC/CGCCGAGAGGGAAGGAG
HAND1	CAAGGATGCACAGTCTGGCGAT/GCAGGAGGAAAACCTTCGTGCT
LHX2	ACGCCAAGGACTTGAAGCAGCT/TTTCCTGCCGTAAGAGGTTGCG
SOX1	GAGTGGAAGGTCATGTCCGAGG/CCTTCTTGAGCAGCGTCTTGGT
RPL27	GGACGCAAAGCTGTCATC/CAATTCCAGCCACCAGAG
RPS29	GCTGTACTGGAGCCACCC/GGCGGCACATATTGAGG
Wild-type AAVS1 locus, without target insert (AAVS1_WT)	CTCTGGCTCCATCGTAAGCAA/CCCAAAGTACCCCGTCTCCC
*M2rtTA* plasmid insert position determination	HA_L-OUT CCGGACCACTTTGAGCTCTAC
	Neo_in-R GCCCAGTCATAGCCGAATAG
*INFB1* plasmid insert position determination	HA_L-OUT CCGGACCACTTTGAGCTCTAC
	Puro_in-R AGGCGCACCGTGGGCTTGTAC
Determination of the presence of non-target inserts of *M2rtTA* plasmid in the genome	M13-R CAGGAAACAGCTATGAC
	Neo_in-R GCCCAGTCATAGCCGAATAG
Determination of the presence of non-target inserts of *INFB1* plasmid in the genome	M13-F GTAAAACGACGGCCAGT
	Puro_in-R AGGCGCACCGTGGGCTTGTAC
Detection of mycoplasma, ribosomal 16S RNA gene	GGGAGCAAACAGGATTAGATACCCT/TGCACCATCTGTCACTCTGTTAACCTC

### 4.6. Spontaneous In Vitro Differentiation

To prove the pluripotency of the obtained iPSC lines, they were subjected to spontaneous differentiation into embryoid bodies with subsequent molecular genetic and immunocytochemical analysis for the main markers of the derivatives of the three germ layers. To obtain embryoid bodies, iPSC colonies were grown on MEFs and were treated with collagenase IV (Gibco™/Thermo Fisher Scientific, Waltham, MA, USA) for 5–10 min at 37 °C; the cells were then mechanically detached using a scraper. The resulting cell aggregates were transferred to non-adhesive conditions (1% agarose-coated plates, Sigma-Aldrich, St. Louis, MO, USA) and were cultured in embryoid body medium: composition until day 15. The medium was changed every three days. After day 15, the formed embryoid bodies were cultured on matrigel-coated glasses for their attachment and subsequent immunofluorescence staining on day 21 or were left in the same non-adhesive conditions for PCR analysis until day 21.

### 4.7. Immunofluorescence Staining

For immunofluorescence staining, the cells were fixed in 4% paraformaldehyde (30 min at room temperature), washed three times with PBS, and incubated with permeabilization and blocking buffer of the following composition: PBS, 10% sheep serum (Sigma-Aldrich, St. Louis, MO, USA), 1% BSA (Sigma-Aldrich, St. Louis, MO, USA), and 1% Triton-X100 (Sigma-Aldrich, St. Louis, MO, USA), for 1 h at room temperature. The cells were then incubated with primary antibodies at 4 °C overnight, were washed extensively three times with PBS solution using an orbital shaker for 20 min at room temperature, and were incubated for 2 h with labeled fluorescent secondary antibodies at room temperature. After the secondary antibodies, the cells were stained with DAPI (BioLegend, San Diego, CA, USA) and immersed into Dako fluorescent medium (Agilent, Santa Clara, CA, USA). The staining was analyzed on a Zeiss LSM 880 confocal microscope (Carl Zeiss, Oberkochen, Germany). Antibodies used for the immunofluorescent staining of iPSC colonies and attached embryoid bodies are listed in Table 2.

### 4.8. Reverse Transcription PCR

For PCR analysis, iPSCs or embryoid bodies were homogenized without prewashing in “ExtractRNA” lysing buffer at room temperature (Evrogen, Moscow, Russia). The obtained lysates were stored at −80 °C or were immediately used to proceed to RNA extraction. Cold chloroform (Komponent-Reaktiv, Moscow, Russia), isopropanol (AppliChem GmbH, Darmstadt, Germany), and 80% alcohol were used for RNA isolation. The isolations were performed according to the “ExtractRNA” protocol. The RNA concentration was determined using a NanoDrop One spectrophotometer (Thermo Fischer Scientific, USA). After isolation, the RNA concentration was equalized for all samples. Reverse transcription was performed using the M-MuLV-RH first-strand cDNA synthesis kit (Biolabmix, Novosibirsk, Russia) according to the manufacturer’s instructions. Real-time PCR was performed using the 5X qPCRmix-HS SyBR-LowROX kit (Evrogen, Moscow, Russia) on a QuantStudio 12K Flex Real-Time PCR System thermal cycler (Thermo Fisher Scientific, Waltham, MA, USA). The results were analyzed using the QuantStudio™ 12K Flex Software v1.2. Ct values were normalized against RPL27 or RPS29 using the 2^−ΔΔCt^ method. Reverse transcription PCR for the detection of *M2rtTA* expression was performed using the 5X ScreenMix PCR mix (Evrogen, Moscow, Russia) under the following conditions: 95 °C—3 min; 38 cycles: 95 °C—15 sec, 62 °C—15 s, 72 °C—30 s; and 72 °C—5 min on a MiniAmp™ Plus Thermal Cycler (Thermo Fisher Scientific, Waltham, MA, USA). After PCR was completed, electrophoresis was performed in a 1% agarose gel using a Mini-Sub Cell GT Cell horizontal phoresis chamber (BioRad, Hercules, California, CA, USA).

The primers used in this study were obtained by DNA-Synthesis LLC, Moscow, Russia. The sequences are listed in Table 1.

### 4.9. Western Blotting

IFN-β expression at the protein level was evaluated in iPSC homogenates and culture supernatants. For supernatant analysis, proteins were first concentrated using the Deoxycholate-trichloroacetic acid precipitation method. Briefly, 50 μL of 0.15% sodium deoxycholate were added to 500 μL of supernatant, and samples were vortexed. After incubation for 10 min at room temperature, proteins were precipitated by adding 50 μL of 100% trichloroacetic acid and incubating on ice for 30 min. Samples were centrifuged at 10,000× *g* for 10 min, and the supernatants were removed. Pellets were washed several times with cold acetone (−20 °C) to remove residual trichloroacetic acid, air dried and resuspended in 70 μL of RIPA buffer (150 mM NaCl, 1.0% NP40, 0.5% sodium deoxycholate, 0.1% SDS, and 50 mM Tris-HCl, pH 8.0, supplemented with Protease Inhibitor Cocktail Set III (Merk, Darmstadt, Germany)). Six microliters of concentrated culture medium per lane was loaded and electrophoresed in a 12% SDS/polyacrylamide gel.

For homogenate analysis, cell pellets were homogenized at 4 °C in RIPA buffer containing Protease Inhibitor Cocktail Set III (Merck, Darmstadt, Germany) and centrifuged at 12,000× *g* for 20 min at 4 °C. Protein concentration was determined using Pierce™ BCA Protein Assay Kit (Thermo Fischer Scientific, New York, NY, USA), and 30 μg of protein was electrophoresed using a 12% SDS/polyacrylamide gel. Human recombinant IFN-β (StemCell Technologies, Vancouver, BC, Canada) was used as a positive control.

After electrophoresis of supernatant- and homogenate-derived proteins, as well as control IFN-β, proteins were transferred onto a 0.45 μm NitroPure™ nitrocellulose membrane (GVS Life Sciences, Bologna, Italy). The membrane was blocked with 5% non-fat dry milk (Cell Signaling Technology, Danvers, MA, USA) in TNT buffer (10 mM of Tris-HCl, pH 7.5, 150 mM of NaCl, 0.1% Tween-20) and incubated overnight at 4 °C with the following primary antibodies in 1% milk: mouse monoclonal anti-human IFN-β (1:1000, #514005, BioLegend, San Diego, CA, USA) and mouse monoclonal anti-β-actin (1:10,000, #A5441 Sigma-Aldrich, St. Luis, MO, USA). The membrane was then washed and incubated for 2 h with HRP-conjugated goat anti-mouse IgG secondary antibodies (1:10,000, #115-035-003 Jackson Immuno Research, West Grove, PA, USA). Chemiluminescent detection was performed using the ECL detection system (Amersham Biosciences, Amersham, UK) and X-ray blue films (Carestream Health, Rochester, NY, USA).

### 4.10. Cultivation of THP-1

A human monocyte-like cell line THP-1 (kindly provided by Dr. V. Tatarskiy) was cultured in RPMI-1640 medium (Biowest, Bradenton, FL, USA) supplemented with 10% FCS (HyClone), 1% penicillin/streptomycin, 1% GlutaMAX, 1% non-essential amino acids (all from Thermo Fisher Scientific) and 0.055 mM β-mercaptoethanol (Sigma-Aldrich). For stimulation experiments, cells were treated with 20 ng/mL of Poly (I:C) (InvivoGen, San Diego, CA, USA) for 24 h prior to RNA isolation.

### 4.11. Statistical Analysis

The data on the graphs are shown as columns with medians and interquartile ranges [25%; 75%] or as boxes and whiskers with minimum and maximum values. The Mann–Whitney test was used for the statistical analysis of differences between groups. A value of *p* < 0.05 was considered to be statistically significant.

## Figures and Tables

**Table 2:** Antibodies used for immunocytochemistry.

	Antibodies	Dilution	Manufacturer, Cat. №
Pluripotency Markers	Rabbit monoclonal anti-OCT4	1:100	Abcam, Cambridge, UK #ab19857
	Rabbit monoclonal anti-SOX2	1:200	Abclonal, Woburn, MA, USA #A11501
	Mouse monoclonal anti-TRA 1-60	1:100	StemCell Techn., Vancouver, BC, Canada, #60064
Differentiation Markers	Mouse Monoclonal Anti-TUBB3	1:200	Abclonal, Woburn, MA, USA A18132
	Rabbit Monoclonal Anti-ACTA2	1:200	Abclonal, Woburn, MA, USA A17910
	Rabbit Polyclonal anti-AFP	1:200	Huabio, Woburn, MA, USA, ER80601
Secondary antibodies	Goat anti-Rabbit IgG, Alexa Fluor 568	1:500	Thermo Fisher Scientific, Waltham, MA, USA #A-11011
	Rabbit anti-Mouse IgG, Alexa Fluor 568	1:500	Thermo Fisher Scientific, Waltham, MA, USA #A-11061

## Data Availability

The original contributions presented in this study are included in the article/Supplementary Materials. Further inquiries can be directed to the corresponding authors.

## Supplementary Materials

The following supporting information can be downloaded at https://www.mdpi.com/article/10.3390/ijms26178376/s1.

## Abbreviations

AFP	alpha-fetoprotein
ACTA2	actin alpha 2, smooth muscle actin
Const-IFNB-iPSCs	iPSC cell lines with constitutively overexpression of interferon-beta
DAPI	4′,6-diamidino-2-phenylindol
Dox	doxycycline hylate
Dox-IFNB-iPSCs	iPSCs with doxycycline- inducible gene overexpression
EBs	embryoid bodies
FCS	fetal calf serum
FGF-2	fibroblast growth factor-2
IFN-I	type I interferon
IFNB1	interferon-beta 1
IFN-β	interferon-beta 1 protein
IFNB-iPSCs	iPSC cell lines with doxycycline-inducible overexpression of interferon-beta
iPSCs	induced pluripotent stem cells
IRF7	interferon regulatory factor 7
K7-iPSCs	K7-4Lf iPSC line (alternative name ICGi022-A)-parent iPSC
MEFs	mouse embryo fibroblasts
M2rtTAf	tetracycline-controlled transactivator protein
Poly (I: C)	polyinosinic-polycytidylic acid
TA-iPSCs	iPSC cell lines with expression of TA
TUBB3	tubulin beta 3.
