# Supplementary material for: The Generation of iPSCs Expressing Interferon-Beta Under Doxycycline-Inducible Control
Source: Int J Mol Sci. 2025 Aug 28;26(17):8376. doi: 10.3390/ijms26178376 (PMC12429120; doi:10.3390/ijms26178376)
Supplement: Supplementary file 1 [file ijms-26-08376-s001.zip › ijms-3750172-supplementary.pdf]

SUPPLEMENTAL DATA

The generation of iPSCs expressing interferon-beta under doxycycline-inducible control

Olga Sheveleva[1*], Nina Butorina[1], Elena Protasova[1], Sergey Medvedev[2], Elena Grigor'eva[2], Victoria Melnikova[3], Valeriia Kuziaeva[1], Marina Minzhenkova[4], Yana Tatarenko[4], Irina Lyadova[1*]

[1] Laboratory of Cellular and Molecular Basis of Histogenesis, Koltzov Institute of Developmental Biology of the Russian Academy of Sciences, Moscow, 119334, Russia
[2] Laboratory of Developmental Epigenetics, Institute of Cytology and Genetics, Siberian Branch of Russian Academy of Sciences, Novosibirsk, 630090, Russia
[3] Laboratory of Comparative Developmental Physiology, Koltzov Institute of Developmental Biology of the Russian Academy of Sciences, Moscow 119334, Russia
[4] Laboratory of Cell biology, Koltzov Institute of Developmental Biology of the Russian Academy of Sciences, Moscow, 119334, Russia

* Correspondence: ivlyadova@mail.ru; Tel.: +7 499-135-87-80
* Correspondence: on_sheveleva@mail.ru; Tel.: +7 499-135-87-80

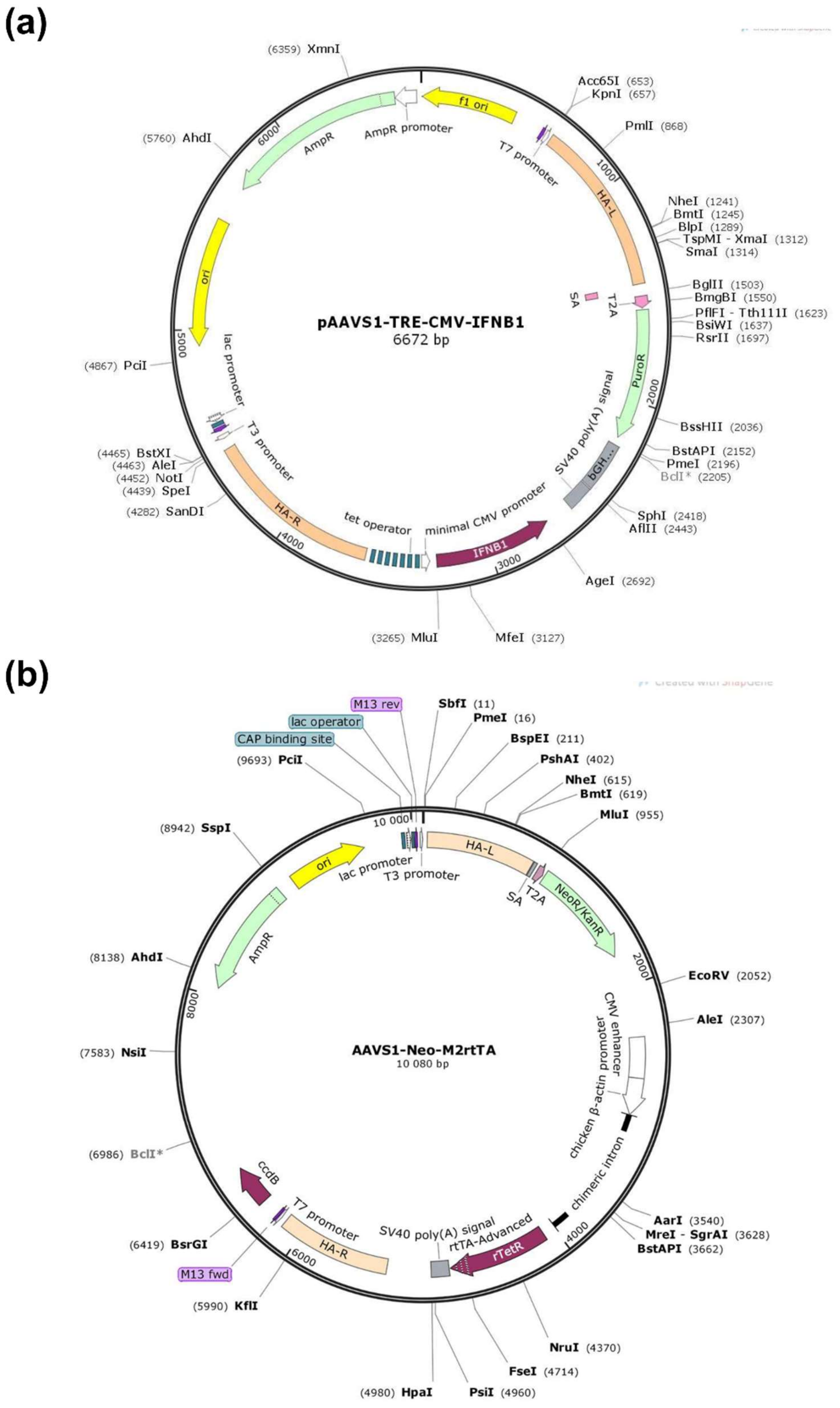

Supplementary Figure S1. Plasmids used for modifying parent K7-iPSCs: pAAVS1-TRE-CMV-IFNB1 (a), containing the target *IFNB1* gene under a doxycycline-driven promoter, and AAVS1-Neo-M2rtTA, containing the *M2rtTA* transactivator (b)

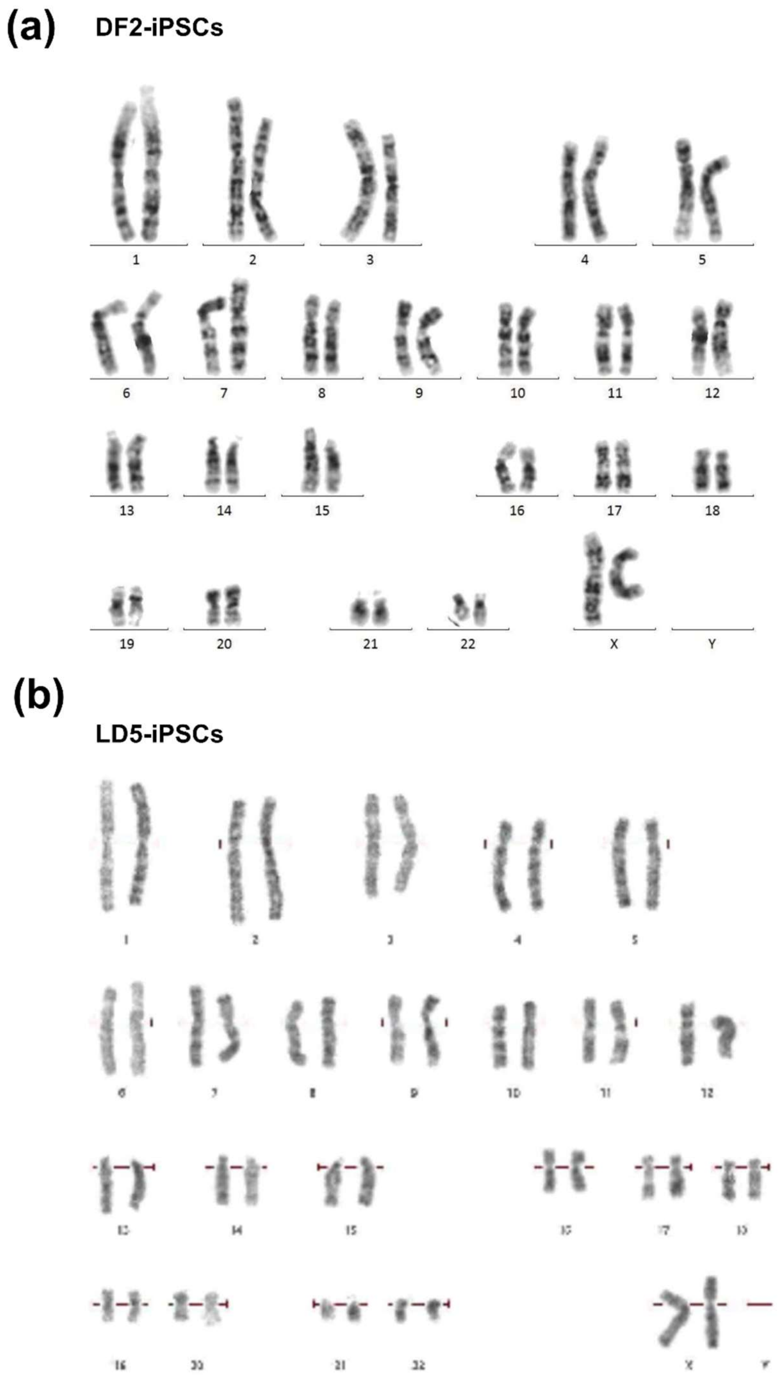

Supplementary Figure S2. Karyograms of DF2-iPSCs and LD5-IPSCs exhibit a normal 46, XX karyotype
